# The Lost Arts

**Published:** 1877-03

**Authors:** 


### EDITORIAL, ETC.

---

*The Lost Arts.*—In the Castellani Collection now on exhibition in the Metropolitan Museum of Art, New York City, are the productions of precious metal workers of prehistoric epochs, the exact dates of which are now unknown. Two kinds of ornaments appear in this valuable collection—those for common use and those for the mourning period—the first being massive and durable and the latter delicate and fragile, but all being of the purest gold.

How the process of melting, soldering and wire drawing which these ornaments exhibit were accomplished, is a mystery which this generation perhaps will not solve. Many attempts have been made to imitate these processes but all have failed, and the mechanical agents employed to separate and join pieces of gold hardly perceptible to the naked eye, and which would be so useful in dental mechanism, have never been discovered.

It is thought to be a question whether fire was employed in this soldering, and the plausible suggestion has been offered that a cold flux was used, which would be an invaluable agent in the dental laboratory, if we only knew how to prepare and apply it.

